# Occurrence, Distribution, and Ecological Risk Assessment of Antibiotics in Different Environmental Media in Anqing, Anhui Province, China

**DOI:** 10.3390/ijerph18158112

**Published:** 2021-07-30

**Authors:** Haiying Chen, Wenfang Zheng, Xiaoming Shen, Fei Zhang, Xiaoping Zhou, Jialin Shen, Ming Lu

**Affiliations:** 1 School of Chemical Engineering, Nanjing University of Science and Technology, Nanjing 210094, China; chaiying@mail.cgs.gov.cn; 2 Nanjing Center, China Geological Survey, Nanjing 210016, China; shenxiaoming@mail.cgs.gov.cn (X.S.); zfei@mail.cgs.gov.cn (F.Z.); zxiaoping@mail.cgs.gov.cn (X.Z.); sjialin@mail.cgs.gov.cn (J.S.)

**Keywords:** antibiotics, Anhui, spatial distribution, ecological risk

## Abstract

The widespread usage of antibiotics in human and animal medication has brought global concerns over environmental contamination of antibiotic residues. In this study, 16 kinds of antibiotics in different environmental media of water, sediments, and soils in Anqing city, Anhui province were determined by ultra-performance liquid chromatography tandem mass spectrometry. A total of fourteen kinds of antibiotics were detected in surface water, with a total concentration up to 479 ng·L^−1^, while six kinds of antibiotics were detected in sediment and soil with concentrations ranging from 15.1 to 108 μg·kg^−1^. Ciprofloxacin (12.8–99.5 ng·L^−1^) and tetracycline (17.2–225 μg·kg^−1^) antibiotics exhibited the highest concentration in water and soil, respectively. In spatial distribution, the total concentration of antibiotics in surface water from the highest to the lowest followed the order of urban area, mainstream of Wan River, suburbs, tributaries of Wan River, indicating that the level of antibiotic concentration in surface water is positively associated with the frequency of human activities. In addition, the antibiotic mass fraction in agriculture land and fishpond were found higher than that in other sampling sites. Moreover, the environmental risk assessment results showed that ciprofloxacin, erythromycin, ofloxacin, enrofloxacin and tetracycline might pose medium to high risks to algae and bacteria in aquatic ecosystem.

## 1. Introduction

In the past decades, antibiotics have been widely used in human and animal medical to prevent and treat bacterial infections [1]. China is the largest producer and user of antibiotics in the world, and there are a large amount of antibiotics distributed in the environment because most of antibiotics are often excreted from body either as original compound or as bioactive metabolites after administration [2]. It was reported that a total of 92,700 tons of antibiotics were used in 2013, among which an estimated 53,800 tons of them eventually entered into the receiving environment [3]. Predictions indicated that global antibiotic consumption in 2030 will be increased up to 200% higher than that in 2015 [4]. At present, antibiotic abuse has received significant worldwide attention, it has led to the rapid up regulation of antibiotic resistance genes, which will pose adverse impacts on humans and other creators in the environment [5,6,7,8,9].

Anhui Province in southeast China is adjacent to the Yangtze River, Huaihe River and Xin’an River Basin. It is a large province of population and antibiotic consumption. There are many investigations reported the occurrence and distribution of antibiotics in rivers and lakes in China [10,11,12,13]. As for Anhui province, Liu [14] and Sun [15] investigated the occurrence of antibiotics in Anhui section of Huaihe River Basin and the aquatic environment in Bengbu city of Anhui Province, respectively. A systematic knowledge on distribution, residue level, sources of antibiotics in different environmental media is beneficial to understand the antibiotic contamination in different regions in Anhui Province. Anqing city is located in the southwest of Anhui Province, and the city’s resident population is 4.72 million. The annual gross domestic product (GDP) of Anqing city is 238.05 billion yuan, ranking fifth in Anhui Province [16]. Wan River, the tributary of the Yangtze River, is Anqing’s mother river. The Yangtze River, which stretches across eastern, central and western China, has a total drainage area of 1.8 million square kilometers [17]. By 2019, the urbanization rate of Anqing has reached 49.98%, with 152.8 million tons of urban sewage and 23.7 million tons of industrial wastewater discharged, and the sewage treatment rate reached 97% [16]. To date, there are no data published regarding the antibiotic contamination in Anqing city.

In this study, surface water, sediment, and soil from different sampling sites in Anqing were collected and analyzed for the antibiotic concentrations, to investigate the occurrence, distribution, and variation of antibiotics. In addition, the potential hazards of current concentration levels of antibiotics in Anqing were explored through assessing the ecological risk of antibiotics in this area. Our study can provide data support and scientific basis for the prevention and control of antibiotic pollution in Anqing city.

## 2. Materials and Methods

### 2.1. Materials and Reagents

A total of 16 typical antibiotics belonging to 5 classes were determined mainly based on their usages in humans and animals in China [18], including sulfonamides (SAs): sulfadiazine (SDZ), sulfamerazine (SMR), sulfadimethoxine (SDM), sulfamethoxazole (SMX), sulfamethazine (SMZ), trimethoprim (TMP); tetracyclines (TCs): oxytetracycline (OTC) and tetracycline (TC); quinolones (QNs): ciprofloxacin (CFX), enrofloxacin (EFX), norfloxacin (NFX) and ofloxacin (OFX); macrolides (MLs): roxithromycin (RTM), clarithromycin (CLR) and a major degradation product of erythromycin (erythromycin-H_2_O, ETM-H_2_O); and lincomycins (LMs): clindamycin (CLIN) were purchased from Rhawn. (Shanghai, China). Isotopically labeled internal standards including sulfadiazine-d4 (SDZ-d4) were obtained from the Laboratory of the Government Chemist (London, UK), tetracycline-d6 (TTC-d6) and roxithromycin-d7 (RTM-d7) were obtained from Sigma-Aldrich Co. (St. Louis, MO, USA), and ciprofloxacin-d8 (CFX-d8) was obtained from Witega (Berlin, Germany). HPLC grade methanol, acetonitrile and formic acid were obtained from Fisher Scientific (Waltham, MA, USA). Disodium ethylenediamine tetraacetate (Na_2_EDTA) was purchased from Sinopharm Chemical Reagent Co., Ltd. (Shanghai, China). Hydrochloric acid (HCl) (guaranteed grade) was purchased from Nanjing Chemical Reagent Co., Ltd. (Nanjing, China). Unless otherwise indicated, the chemicals used were analytical grade or above.

The sample pretreatment was conducted according to a previously reported method [18,19]. The target antibiotics were analyzed by ultra-high performance liquid chromatography (UPLC, Thermo Ultimate, Waltham, MA, USA) coupled with a triple quadrupole mass spectrometer (MS, Thermo TSQ Quantum Access MAX, USA) equipped with an electrospray ionization (ESI) source in multiple reaction monitoring (MRM) mode. A total of 10μL of the redissolved extract was injected into the chromatographic system. The target antibiotics were separated by a C18 column (2.1 mm × 100 mm, 5 μm, Thermo, Waltham, MA USA) maintained at 35 °C. The flow rate of gradient elution was 0.25 mL/min with Phase A (Milli-Q water with 0.1% (*v*/*v*) formic acid) and Phase B (methanol). The separation of antibiotics was achieved with a gradient program as follows: 0–2.2 min, 16% B; 2.2–2.5 min, 16% B–95% B; 2.5–5.5 min, 95% B; 5.5–6.0 min, 95% B–16% B; 6.0–10.0 min, 16% B.

### 2.2. Standard Solution Preparation

The reference antibiotics and internal standard were dissolved in methanol to prepare the standard stock solution with a mass concentration of 100 mg·L^−1^. The stock solution was diluted to 1 mg·L^−1^ with methanol before use, and all the standard solutions were kept in a refrigerator at −20 °C in dark.

### 2.3. Sampling Site Description and Sample Collection

The center of Anqing City is located between 29°47′–31°16′ N and 115°45′–117°44’ E, including Yingjiang, Daguan and Yixiu districts. Sampling sites were set up in all three districts, among which 7 sampling sites were set in Daguan District, because the Wan River flows through the area and then flows into the Yangtze River. One sampling point was set in Yingjiang District with the highest urbanization rate, and another sampling site was set in the orchard of Yixiu District with the highest forestry output value. The detailed information of study region was provided in Table S1.

**Figure 1:**
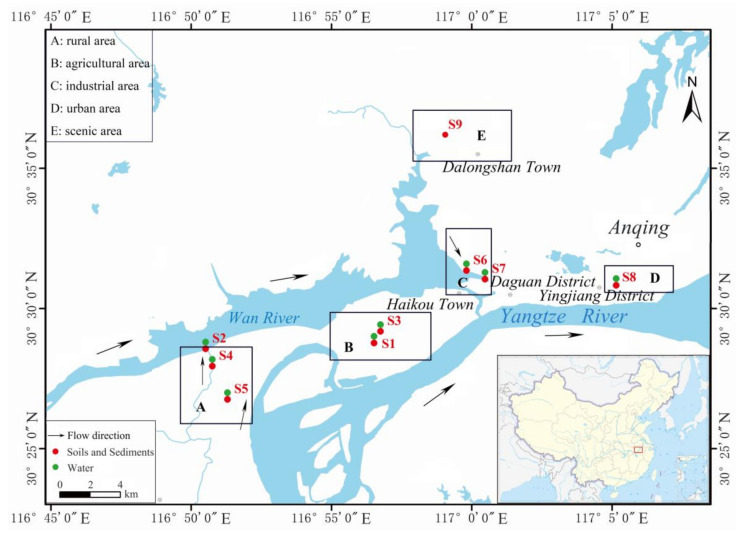
Cartographic illustration of the sampling sites in Anqing city.

As shown in Figure 1, samples S1–S7 (covering rural areas, industrial areas, and agricultural areas) were collected along the upper, middle and lower reaches of WanRiver Basin in Daguan District. Sample S8 was collected in Yingjiang District (urban area), and sample S9 was collected in Yixiu District (scenic area).Surface water (*n* = 8), sediment (*n* = 5) and soil (*n* = 4)samples were collected during the sampling campaigns in October 2020. Administrative region information and types of land of sampling sites were provided in Table S2.

Sampling sites S2, S4 and S5 are located in the upper reaches of the Wan River in rural areas with low population density. Among them, S5 is located near the farm, S4 is located in the tributary of Wan River, and S2 is located in the mainstream of Wan River. Sampling sites S1 and S3 are located in the agricultural area of the middle reaches of Wan River, with relatively dense population. Among them, S1 is the vegetable farmland, and S3 is the fish pond, mainly raising fish, shrimp and other aquatic products. Sampling sites S6 and S7 are located in the high-tech industrial development district of Anqing in the lower reaches of Wan River. The Anqing High-Tech Zone is an important petrochemical industry base in China and a new chemical material industry base in Anhui Province, among which chemical plants are located near S7. S8 is located in the main urban area of Anqing, where the population is very dense. The sampling site is near the urban sewage treatment plant. Sampling site S9 is located in Dalongshan Town in the northern suburb of Anqing, near the famous Dalongshan Scenic Spot. The S9 sampling site is located in the orchard here, less affected by human. Detailed information was shown in Table 1.

Water and soil samples (0–20 cm) were collected using stainless steel sampler, sediment samples (0–10 cm) were collected by stainless-steel grab sampler at the corresponding water sample location [20,21,22]. The water samples were stored in a 1 L brown glass bottle, with 3 parallel samples in each group. The soil and sediment sam-ples were stored in 250 mL wide-mouth brown glass bottles. Three subsamples were collected at each soil and sediment sampling site, which were combined into a composite sample. All samples were stored in dark at 4 °C and taken back to the laboratory within 24 h for further pretreatment. Sediment and soil samples were freeze-dried, ground, and sieved through a 0.5-mm pore size, then stored in a −20 °C freezer until extraction.

### 2.4. Quality Assurance and Quality Control

To ensure the quality of analysis, laboratory quality assurance and quality control methods (QA/QC) were implemented, including the method quantification limits (MQLs), solvent blanks, method blanks, surrogate usage, recovery of spiked samples, and analysis of replicates. The recovery experiments (*n* = 3) were conducted in different matrix of water, soil, and sediments. The recovery ranges of antibiotics in water, soil and sediments were 71–108%, 62–115%, and 65–117%, respectively. The relative standard deviation (RSD) percentages of the spiked measurements in water, soil and sediments were 1.2–13.3%, 2.2–25.6%, and 2.7–23.8%, respectively. The MQLs was 0.18–3.25 ng·L^−1^ for water samples, 0.35–6.50 μg·kg^−1^ for soils and sediments. All samples were set up with 3 parallel samples to obtain the mean concentration. Internal standard method was used to calculate the concentrations of target compounds. To confirm the reliability of the instrument, solvent blank, procedural blank and known standard were injected every 15 samples during the instrumental analysis.

### 2.5. Ecological Risk Assessment of Antibiotics

According to the EU risk assessment technical guidance document, the ecological risk of antibiotics in water can be assessed by risk entropy.

RQ = MEC/PNEC(1)

PNEC = TD/AF(2)

RQ (risk quotient), MEC (measured environmental concentration), PNEC (predicted no-effect concentration), TD (toxicity data, acute or chronic toxicity data for existing studies), and AF (assessment factor). AF value was 100 if chronic toxicity data were used, and AF value was 1000 if acute toxicity data were used [23]. Based on the most unfavorable situation, the most sensitive aquatic species were generally used to calculate the RQ value. The RQ results were classified as low, medium, and high risk. The interpretation of RQ is: RQ ≥ 1 for high risk, 0.1 ≤ RQ < 1 for medium risk, and RQ < 0.1 for low risk [19]. The toxicity data of 16 antibiotics in this study were mainly cited from the previously published data, which were shown in Table 2.

## 3. Results

### 3.1. Occurrence of Antibiotics in Anqing

The detailed concentrations and detection frequency of antibiotics in Anqing city were shown in Table 3. Among the 16 antibiotics, the detection frequency of TC and OTC were up to 100%, while SMX and SMZ were not detected in any sample.

As shown in Table 3, 14 antibiotics were detected in surface water, among which the detection frequency of 8 antibiotics (CFX, NFX, OFX, EFX, TC, OTC, SDZ and RTM) reached 100%, indicating that antibiotics are widely existed in this area. TC (17.2–225 ng·L^−1^, mean 63.1 ng·L^−1^) and CFX (17.2–225 ng·L^−1^, mean 42.9 ng·L^−1^) were the top two antibiotics in water, and contributed 32.2% and 21.8% of the total antibiotic concentration, respectively.

As for the sediment/solid, the detection frequency and concentrations of target antibiotics indicated that 6 antibiotics of TC, OTC, NFX, CCR, CFX and ETM were detected in sediment/solid, with the mean mass fraction of 23.3, 8.60, 2.81, 2.26, 1.28 and 1.19 μg·kg^−1^, respectively. It is worth pointing out that SAs were not detected in our collected solid samples. Among the detected 6 antibiotics, TC (ND-43.3 μg·kg^−1^) and OTC (ND-24.3 μg·kg^−1^) exhibited much higher mass fraction than the other four antibiotics.

### 3.2. The Spatial Distributionof Antibiotics in Anqing

**Figure 2:**
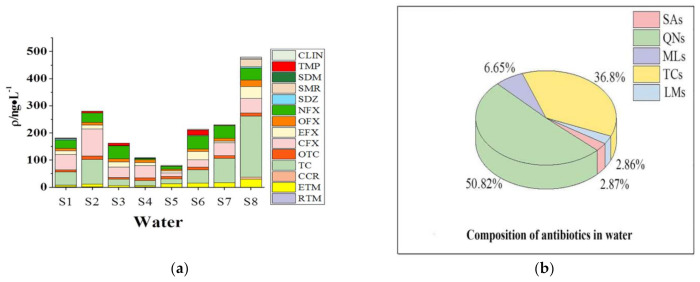
(**a**): Distribution of antibiotics in surface water in Anqing; (**b**): composition of antibitics in water samples.

As shown in Figure 2b, QNs and TCs had higher detection frequency in water, and contributed 50.8% and 36.8% of the total antibiotic concentration, respectively. As shown in Figure 2a, the total antibiotic concentration at site S5 was the lowest, while that at site S8 was the highest. The total concentration of the collected 8 samples from the highest to the lowest, followed the order of S8 (479 ng·L^−1^) > S2, S6 and S7 (200–300 ng·L^−1^) > S1 and S3 (150–200 ng·L^−1^) > S4 and S5 (about 100 ng·L^−1^). Sample S8 was collected from the WWTP area, and the pollution sources may be domestic sewage. Many studies demonstrated that WWTP effluent is an important source of antibiotics in surface water [28,29], because antibiotics are widely used in China, and a large amount of residues would be discharged into the aquatic environments due to their limited removal in WWTPs [3]. Samples S2, S6 and S7 were all collected from the mainstream of Wan River, and the total antibiotic concentration for each sample was quite similar. The chemical plant close to S7 sampling site was not found to show obvious impact on the antibiotic concentration. Samples S1 and S3 (collected from suburbs section) were found to have low levels of antibiotic concentration. Although there is a small family farm with dozens of pigs and thousands of chickens near sampling site S5, the low antibiotic concentration implied that the farm had no impact on the antibiotic concentration in surface water. The total antibiotic concentration in different function areas from the highest to the lowest followed the order of urban area, Wan River mainstream, suburbs and Wan River tributaries, indicating the antibiotic concentration was positively associated with the frequency of human activities [3].

**Figure 3:**
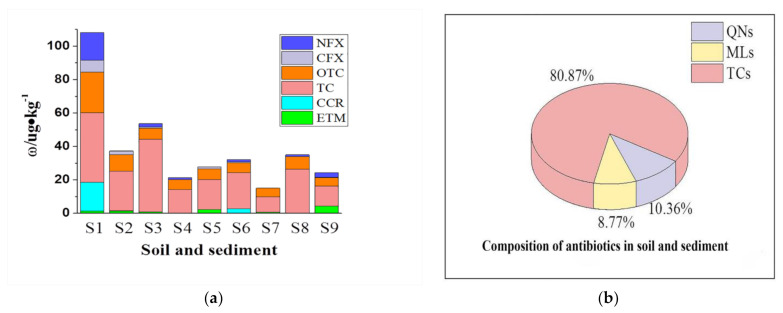
(**a**): Distribution of antibiotics in soil/sediment in Anqing; (**b**): composition of antibiotics soil/sediment samples.

As shown in Figure 3b, TCs and QNs had higher detection frequency in sediment/soil, and contributed 80.9% and 10.4% of the total antibiotic concentration, respectively. As for the spatial distribution, the agricultural land (S1) and aquaculture pond (S3) were more likely to have higher antibiotic mass fraction level (seen in Figure 3a). The total antibiotic mass fraction at sampling site S1 was the highest, with up to 108 μg·kg^−1^, and the existing of antibiotics in this area is probably due to animal manure, which was recognized as one of the two main pathways of antibiotic transfer in various environmental compartments [30]. The total mass fraction of antibiotics for S3 was 53.6 μg·kg^−1^, in which TC (43.3 μg·kg^−1^) accounted for a large proportion. Sample S3 was collected from fish pond, which was mainly for cultivation of fish, crabs, and shrimp.TC was frequently used to control gastrointestinal disorders and respiratory problems in aquaculture pond [7]. Except for S1 and S3, other sampling sites showed a lower antibiotic mass fraction levels of below 40 μg·kg^−1^.The spatial distribution in soil and sediment from the highest to the lowest followed the order of agricultural land, fish pond and others.

### 3.3. Ecological Risk Assessment of Antibiotics

**Figure 4:**
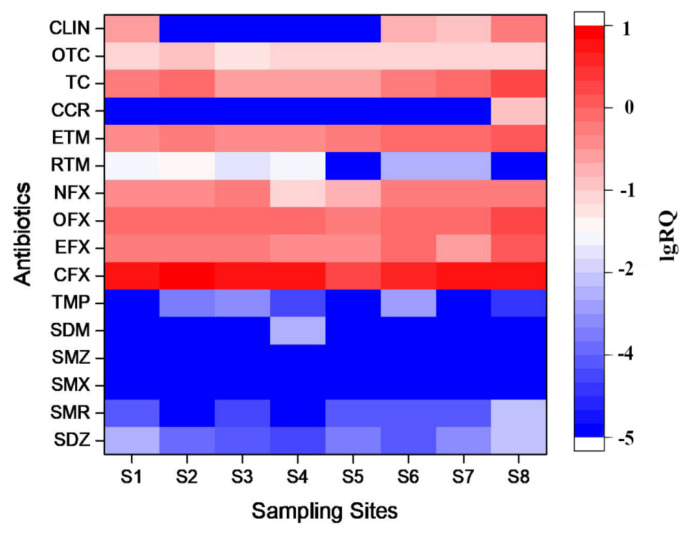
Ecological risk assessment of antibiotics in surface water.

In order to understand the risk levels of antibiotics in Anqing, the risk assessment results of this study were analyzed by lgRQ [18]. The results were illustrated in Figure 4. It is worth noting that the lgRQ values of CFX, EFX, OFX, TC and ETM-H_2_O at all sampling sites were greater than −1, indicating medium to high risks. The samples proportions classified as high risk were 100% for CFX, 37.5% for TC, 25.0% for EFX and OFX and 12.5% for ETM, respectively. It was likely because the lowest EC_50_ values of these antibiotics in the toxicological experiments (Table 2) were used in the risk assessment, which represented the most sensitive species. In 87.5% and 37.5% of surface water samples, NFX and CLIN may cause medium risk. The lgRQ values of RTM, CCR and OTC were less than−1, suggesting a low risk. The lgRQ values of SAs and TMP were less than −2 in all sampling sites, indicating that the ecological risks of these antibiotics were very low.

Overall, among the 16 target antibiotics, 7 exhibited potential varying degree ecotoxicological risks in the waters due to their high concentrations. Particularly, CFX, EFX, OFX, TC and ETM-H_2_O in this area exhibited relatively high ecological risk to the relevant sensitive aquatic organisms. Similar results were also reported in Wangyang River where TC, ETM, OFX and CFX levels showed relatively high ecological risks to algae and bacteria in aquatic ecosystem [24].

## 4. Discussions

### 4.1. Comparison of Antibiotic Contamination Characteristics in Water and Soil/Sediment

In terms of the number and detection rate of antibiotics, 14 antibiotics detected in water were much more than 6 in soil/sediment (Table 3). From the perspective of antibiotic content, their mean concentrations in water and soil/sediment decreased in the following orders: QNs > TCs > MLs > SAs > LMs and TCs > QNs > MLs respectively (Table 3). It was found that antibiotics with high concentrations in water are also relatively high in soil, and their pollution is relatively similar, such as QNs, TCs and MLs. MLS and SAs are very low in water and difficult to adsorb in sediment and soil, so they are not detected in soil/sediment. Usually, TCs have been rarely detected in natural water because it has strong degradation tendency in natural water and is easily adsorbed on solid surface [31]. However, the detection frequency and concentration of TC were higher than that of other antibiotics in Anqing, which was in accordance with previous result in Wangyang River in northern China [24], suggesting the large consumption and discharge of TC in this area. Expectedly, QNs and TCs were the dominant pollution factors in sediment/soil, which were similar to those in Xiangjiang River [32] and Yangtze Estuary [33].

### 4.2. Comparison of Antibiotics in Surface Water of Different Aquatic Environment in China

As can be seen in Table 4, the types and concentrations of antibiotics in the surface water vary with the regions in China. Generally, antibiotic concentrations in surface water in Anqing were almost at a lower or moderate level compared with that in other regions in China, which was presumably due to less pollutant sources, high temperatures and heavy summer rainfall in the lower-middle reaches of the Yangtze River Basin [34]. Moreover, most antibiotics exhibited a similar concentration in Anqing to those in Anhui section of Huaihe River Basin [14], whereas much lower than some other rivers in China (i.e., Liao River, Yellow River, Liao River). QNs (4.16–99.5 ng·L^−1^) and MLs (ND-15.9 ng·L^−1^) exhibited slightly higher concentration in Anqing than that in Songhua River.

### 4.3. Comparison of Antibiotics in Sediment/Soil of Different Aquatic Environment in China

As can be seen in Table 5, QNs and TCs antibiotics in river sediments (such as: Hai Rive [18], Liao River [36]) were almost two to three orders of magnitude higher than those in Anqing. It was also noticed that antibiotics in sediment in Anqing were slightly lower than those in the Songhua River [35] and Yellow River [38], whereas TCs in sediment in Anqing were slightly higher than those in Taihu Lake [40]. In addition, the residues in soil in Anqing were lower almost an order of magnitude than those in four provinces of China (Hebei, Henan, Sichuan, Jiangsu) [41]. In general, antibiotic concentrations in sediment and soil in Anqing were almost at a lower level.

## 5. Conclusions

This study investigated the contamination characteristics and ecotoxicological risks of antibiotics in Anqing city, Anhui Province, by analyzing16 typical antibiotics in surface water, soil and sediment. The results showed that:

(1). Approximately 80.0% of the individual antibiotic concentration were lower than 20.0 ng·L^−1^ in water and 85.2% of the individual antibiotic mass fraction were lower than and 5.0 μg·kg^−1^ in soil and sediment. QNs and TCs were the predominant detected antibiotics at all sampling sites. Compared with the environment of other regions in China, the antibiotic concentration in Anqing was generally at a low level.
(2). The distribution of total antibiotics in surface water varied with space, while the distribution of total antibiotics in the soil and sediment in different sampling sites were not significantly different in Anqing. The direct discharge of domestic wastewater, the livestock and aquaculture sewage were considered as the dominant sources of antibiotics. The human activity frequency was closely related to the degree of antibiotic pollution.
(3). From the ecological risk perspective, CFX exhibited significant acute toxicity risks for algae, which was at high risk level. EFX, OFX, TC and ETM-H_2_O were at medium to high risk level. Based on the ecological risk of individual antibiotic, five representative antibiotics (CFX, EFX OFX, TC and ETM-H_2_O) were screened out from a wide range of species for antibiotic monitoring in this area.

## Figures and Tables

**Table 1:** Detailed information of sampling sites in Anqing city.

Sampling No.	Sampling Sites	Area	Sample Type
S1	agricultural land	suburbs	water/soil
S2	upstream	mainstream of Wan River	water/sediment
S3	fish pond	suburbs	water/sediment
S4	tributaries	tributaries of Wan River	water/sediment
S5	farm	tributaries of Wan River	water/soil
S6	downstream	mainstream of Wan River	water/sediment
S7	near chemical plant	mainstream of Wan River	water/sediment
S8	near WWTP	urban area	water/soil
S9	orchard	suburbs	soil

**Table 2:** Toxicity data of antibiotics, AF value and PNEC value.

Antibiotics	Algae	Toxicity Data (mg·L^−1^)	AF	PNEC (ng·L^−1^)	References
SDZ	Selenastrum capricornutum	EC_50_ = 2.2	1000	2200	[24]
SMR	Scenedesmus vacuolatus	EC_50_ = 11.9	1000	11,900	[25]
SMX	Synechococcus leopoliensis	EC_50_ = 0.027	1000	27	[24]
SMZ	Scenedesmus vacuolatus	EC_50_ = 19.52	1000	19,520	[24]
SDM	Lemna minor	EC_50_ = 0.248	1000	248	[26]
TMP	Rhodomonas salina	EC_50_ = 16	1000	16,000	[24]
CFX	Microcystis aeruginosa	EC_50_ = 0.005	1000	5	[24]
EFX	Vibrio fischeri	NOEC_50_ = 0.00288	100	28.8	[24]
OFX	Pseudokirchneriella subcapitata	NOEC_50_ = 0.00113	100	11.3	[24]
NFX	Vibrio fischeri	NOEC_50_ = 0.01038	100	103.8	[24]
RTM	Pseudokirchneriella subcapitata	NOEC_50_ = 0.01	100	100	[24]
ETM-H_2_O	Pseudokirchneriella subcapitata	EC_50_ = 0.02	1000	20	[24]
CCR	Pseudokirchneriella subcapitata	EC_50_ = 0.23	1000	230	[27]
TC	Microcystis aeruginosa	EC_50_ = 0.09	1000	90	[24]
OTC	Microcystis aeruginosa	EC_50_ = 0.207	1000	207	[24]
CLN	Pseudokirchneriella subcapitata	NOEC_50_ = 0.014	1000	14	[27]

**Table 3:** Concentrations and frequency of antibiotics in Anqing.

Antibiotics	Surface Water (*n* = 8, ng·L^−1^)			Sediment/Soil (*n* = 9 μg·kg^−1^)		
	Range	Mean	Frequency (%)	Range	Mean	Frequency (%)
SDZ	0.20–5.77	2.26	100	ND	ND	0
SMR	ND-27.4	4.57	75	ND	ND	0
SMX	ND	ND	0	ND	ND	0
SMZ	ND	ND	0	ND	ND	0
SDM	ND-2.31	0.37	12.5	ND	ND	0
TMP	ND-20.0	4.76	62.5	ND	ND	0
CFX	12.8–99.5	42.9	100	ND-6.94	1.28	66.7
EFX	6.88–43.6	17.2	100	ND	ND	0
OFX	6.72–24.5	11.1	100	ND	ND	0
NFX	4.16–48.6	30.4	100	ND-16.7	2.81	77.8
RTM	ND-1.16	1.20	75	ND	ND	0
ETM-H_2_O	6.35–29.5	12.3	100	ND-4.29	1.19	66.7
CCR	ND-7.82	0.98	12.5	ND-17.3	2.26	44.4
TC	17.2–225	63.1	100	ND-43.3	23.3	100
OTC	6.94–13.5	9.65	100	ND-24.3	8.60	100
CLIN	ND-6.42	1.76	50	ND	ND	0

ND, not detected.

**Table 4:** The concentration of antibiotics in surface water in China (ng·L^−1^).

Regions	Sampling Time	Antibiotic Types and Concentrations					Reference
		SAs	QNs	MLs	TCs	LMs	
Songhua River	2017.10	ND-26.9 (14.8)	ND-7.1 (2.6)	ND-6.9 (3.8)	—	—	[35]
Liao River	2015.7–11	ND-56.4 (25.4)	10.2–441.7 (137.8)	17.4–496.5 (151.4)	ND-849.7 (187.1)	—	[36]
Hai River	2010.09	27.4–317 (187)	26.5–196 (121)	6.6–33.4 (17.1)	—	—	[37]
Yellow River	2014.09	—	54.79–173.66 (82.65)	4.7–27.64 (12.71)	32.71–131.59 (49.79)	—	[38]
Yangtze River	2013.autum	40.3–310.7	—	11.9–125.7	—	—	[39]
Taihu Lake	2015.12	8.7–34.7 (16.6)	17.4–57.7 (29.7)	25.6–89.1 (60.5)	69.8–189.6 (112)	—	[11]
Huaihe River	2018.12	6.2–19	—	—	5.7–170	—	[14]
This study	2020.10	ND-27.43 (1.95)	4.16–99.51 (25.39)	ND-15.90 (4.83)	6.94–225 (36.67)	ND-6.42 (1.76)	

ND, not detected; ( ) means average value. The same below.

**Table 5:** Comparison of antibiotics in sediments/soil (ng·g^−1^) in Anqing with those found in other environments.

Regions	Sample	Antibiotics Types				Reference
		SAs	QNs	MLs	TCs	
Song Hua River	sediment	ND	0.8–117.9(41.5)	3.0–28.3(11)	—	[35]
Liao River	sediment	ND-6.1(5.8)	ND-640(230.3)	ND-78.8(27)	ND-512(186.8)	[36]
Hai River	sediment	(1327.4)	(1644.4)	(291.8)	(2783.2)	[18]
Yellow River	sediment	—	27.49–20 (73.98)	2.75–7.29(4.15)	4.4–38.27(14.65)	[38]
Taihu lake	sediment	ND-2.45(0.35)	ND-80.4(12.2)	ND-3.56(0.39)	ND-39.6(4.12)	[40]
four provinces	farm soil	ND-15.39(2.61)	ND-141(12.78)	ND-83.04(12.24)	ND-415(82.75)	[41]
This study	sediment	ND	ND-2.10(0.40)	ND-2.73(0.38)	5.14–43.3(14.6)	
This study	soil	ND	ND-16.7 (1.80)	ND-17.3 (2.11)	5.20–41.6 (17.6)	

## Data Availability

The data used to support the findings of this study are available from the corresponding author upon reasonable request.

## Supplementary Materials

The following are available online at https://www.mdpi.com/article/10.3390/ijerph18158112/s1, Table S1: Basic information of the studied regions of Anqing in 2020, Table S2: Administrative region information and types of land of sampling sites.
