# Supplementary material for: Occurrence, Distribution, and Ecological Risk Assessment of Antibiotics in Different Environmental Media in Anqing, Anhui Province, China
Source: Int J Environ Res Public Health. 2021 Jul 30;18(15):8112. doi: 10.3390/ijerph18158112 (PMC8346025; doi:10.3390/ijerph18158112)
Supplement: Supplementary file 1 [file ijerph-18-08112-s001.zip › ijerph-1296013-supplementary.pdf]

International Journal of
Environmental Research and Public Health

MDPI

Article

Occurrence, Distribution, and Ecological Risk Assessment of Antibiotics in Different Environmental Media in Anqing, Anhui Province, China

Haiying Chen [1,2], Wenfang Zheng[1,*] and Xiaoming Shen [2], Fei Zhang [2], Xiaoping Zhou [2], Jialin Shen [2] and Ming Lu [1,*]

[1] School of Chemical Engineering, Nanjing University of Science and Technology, Nanjing 210094, China; chaiying@mail.cgs.gov.cn
[2] Nanjing Center, China Geological Survey, Nanjing 210016, China; shenxiaoming@mail.cgs.gov.cn (X.S.); zfei@mail.cgs.gov.cn (F.Z.); zxiaoping@mail.cgs.gov.cn (X.Z.);sjialin@mail.cgs.gov.cn(J.S.)
* Correspondence: zhwf@njust.edu.cn (W.Z.); luming@njust.edu.cn (M.L.)

Citation: Chen, H.; Zheng, W.; Shen, X. Occurrence, Distribution, and Ecological Risk Assessment of Antibiotics in Different Environmental Media in Anqing, Anhui Province, China. *Int. J. Environ. Res. Public Health* **2021**, *18*, 8112. https://doi.org/10.3390/ijerph18158112

Academic Editors: Giulia Simonetti, Francesca Buiarelli and Paul B. Tchounwou

Received: 28 June 2021
Accepted: 23 July 2021
Published: 30 July 2021

Publisher's Note: MDPI stays neutral with regard to jurisdictional claims in published maps and institutional affiliations.

Copyright: © 2021 by the authors. Licensee MDPI, Basel, Switzerland. This article is an open access article distributed under the terms and conditions of the Creative Commons Attribution (CC BY) license (http://creativecommons.org/licenses/by/4.0/).

Table S1. Basic information of the studied regions of Anqing in 2020.

Section	Area (Km^2)	Population of the year end (10^4 persons)	Population density (person /km^2)	Urbanization (%)	Output Value of agriculture (10^4 yuan)	Output Value of Forestry (10^4 yuan)	Output value of animal husbandry (10^4 yuan)	Output value of fishery (10^4yuan)
Anqing	13538	472.3	349	50.0	1607723	357004	1153133	636765
Yingjiang District	207	27.7	1338	87.0	24143	331	4956	5441
Daguan District	235.5	29.5	1253	67.7	51813	4902	35125	32166
Yixiu District	414	28.2	681	46.9	38170	10462	36356	49035

Note: Data from Anqing and three urban districts, and all data were obtained and calculated from Anqing Statistical Yearbook, 2020. (http://tjj.anqing.gov.cn/tjsj/tjnj/index.html).

Table S2. Administrative region information and types of land of sampling sites.

Sampling No.	Sampling sites	Types of land	Administrative region
S1	agricultural land	agricultural area	Haikou Town,Daguan District
S2	upstream	rural area	Juwang Village, Haikou Town, Daguan District
S3	fish pond	agricultural area	Haikou Town, Daguan District
S4	tributaries	rural area	Yisheng Village, Haikou Town,Daguan District
S5	farm	rural area	Hongsheng Village, Haikou Town, Daguan District
S6	downstream	industrial area	High-tech Zone, Daguan District
S7	near chemical plant	industrial area	High-tech Zone, Daguan District
S8	near WWTP	urban area	Yingjiang District
S9	orchard	scenic spot	Dalongshan town, Yixiu District
